# Experimental analysis of rotating bridge structural responses to existing railway train loads via time–frequency and Hilbert–Huang transform energy spectral analysis

**DOI:** 10.1038/s41598-024-58795-0

**Published:** 2024-04-10

**Authors:** Xu Liu, Honggang Wu, Shouquan Zhao, Xuehu Yang

**Affiliations:** 1 College of Resources and Environmental Engineering, Guizhou University, Guiyang, 550025 China; 2 China Northwest Research Institute Co., Ltd of C.R.E.C, Lanzhou, 730030 China; 3 Lanzhou Jiaotong University, Lanzhou, 730070 China

**Keywords:** Time–frequency, HHT energy spectrum, Existing line, Rotating bridge, Vibration waves, Marginal spectrum, Engineering, Civil engineering

## Abstract

With the rapid development of national infrastructure projects, there has been a significant increase in intersecting lines in transportation construction. As a result, rotating bridges are increasingly used in engineering projects that span existing railway lines. In order to study the spatial response characteristics and vibration wave transmission mechanisms of the rotating bridge structure under the loading of existing railway trains, field experiments and numerical analyses were conducted. The response characteristics of these bridges were investigated under different types and speeds of adjacent existing lines. A comprehensive methodology has been proposed, integrating the time domain spectrum and the Hilbert–Huang Transform (HHT) energy spectrum for signal processing and vibration analysis. The analysis was carried out using MATLAB 2018a software. This methodology was applied to analyze the test data. The results show that significant resonance phenomenon occurs in the girders of the rotating bridge under the loading of trains on the existing line. The low-frequency component *f*_1_ (2–5 Hz) is the primary factor contributing to the amplification of the acceleration response in the rotating bridge, while *f*_3_ (10–13 Hz) plays a secondary role. The frequency distribution characteristics of vibration waves caused by train loads on the existing line have a significant influence on the acceleration response of the rotating bridge's girders. The predominant frequency of vibration waves at each measuring point along the transmission path shows a trend of decreasing → increasing → decreasing. The impact on the rotating bridge structure of vibration waves generated by low-speed freight trains on existing railways is greater. The research findings are of great importance for studying the dynamic response of rotating bridges adjacent to existing railway lines.

## Introduction

In recent years, the focus has shifted towards the high-quality development of the western regions, driven by the rapid growth of infrastructure development in China. This has provided new impetus for implementing the Western Development Strategy and achieving the goal of building a national transportation powerhouse. Significant progress has been made in high-speed rail construction, including the completion of the “Four Vertical and Four Horizontal” high-speed rail network^1,2^. Efforts are currently underway to advance the construction of the “Eight Vertical and Eight Horizontal” high-speed rail network. As a result of this rapid expansion of the transportation network, there has been an increase in the number of engineering projects, including the construction of new bridges spanning existing railways. To ensure the seamless operation of existing railway lines and simplify construction complexity, rotating bridge construction schemes have become commonplace^3,4^. These bridges are constructed by working on the ground parallel to the existing railway lines on both sides, building the bridge’s main piers and upper beam structures. After completing all procedures, the bridge span structure is rotated to the axis position over the railway line using traction equipment. Finally, the bridge is joined seamlessly, forming an overpass over the existing railway line. However, the train loads of the existing lines pose non-negligible threats to the construction of rotating bridges, especially in terms of structural accuracy. Therefore, it is necessary to carry out research on the structural response law and the transmission mechanism of the vibration wave of the rotating bridge under the action of the train load of the existing line.

The technique of bridge rotation was first applied and developed in the 1940s^5–7^. The introduction of this technology has been instrumental in facilitating the construction of bridges over railways, canyons, rivers and oceans. However, to date, the majority of research into rotating body bridges has focused on numerical simulations and laboratory scale model experiments. Zhang^8^ introduced and applied the seismic analysis method to rotating bridges, demonstrating the feasibility of implementing turning maneuvers in large cantilever level transition rotating bridges without disrupting traffic. This was achieved by comparing measured data with finite element simulation results. Sun et al.^9^ conducted simulations to model the vertical vibration load induced by trains using an excitation force function, specifically in the context of an actual project. They investigated the vibration response characteristics of the turning structure under the influence of train loads. Wang et al.^10^ employed finite element analysis to assess the dynamic response of the rotating bridge structure subjected to pulsating wind effects. They conducted a novel evaluation of the impact of pulsating wind on the structural stability using the radial variation index of ball-hinged compressive stress. Wang et al.^11^ established the first three-dimensional numerical simulation model of a single-point flat-hinged transcontinental cable-stayed bridge and analyzed the force characteristics of each member of the transcontinental cable-stayed bridge during the transcontinental process. Zhang et al.^12^ proposed a real-time method to assess the structural stability during horizontal rotation by measuring the vibration acceleration of the rotating structure. The developed analytical formula accurately captures the relationship between vibration acceleration and pier-bottom bending moment, ensuring the safety of bridges during the rotation process. Liu et al.^13^ proposed a novel model, based on non-Hertz contact theory, for calculating the critical overturning moment of a rotating bridge. The primary objective of this model is to enhance safety during the rotational construction process of such bridges. Furthermore, related scholars have conducted a range of studies investigating the impact of train loads on the response of adjacent structures along existing railway lines. Meng et al.^14^ developed a three-dimensional model of the subway tunnel-foundation-existing bridge using the finite element software ABAQUS. They integrated this model with a simplified mechanical model to study the structural response of the adjacent bridge to train-induced vibrations. The study revealed significant vibrations in the bridge structure, but no diffusive vibration effect was observed, and the amplitude of vibrations was small. Degrande et al.^15^ analyzed the response of the free field, the track and the surrounding buildings during the passage of trains with different speeds by means of field measured data. It was found that in the free field, the horizontal and vertical vibrations generated during train passage have similar amplitude magnitudes, and the peak particle velocity decreases with increasing distance from the tunnel and is only weakly related to the train speed. Vogiatzis^16^, Ma et al.^17^, Zhu et al.^18,19^, on the other hand, studied the effects of subway train loads on the dynamic vibration of ancient building structures by means of numerical simulations and field measurements, which provided relevant references for the design and vibration load control of existing lines around ancient buildings.

Rotating bridges are subject to stochastic excitation during construction and are influenced by environmental factors such as wind loads. In addition, the dynamic response of train loads in the vicinity of existing railway lines exhibits characteristics such as small amplitude, high randomness, nonlinearity and unsteady signals. Compared to the traditional Fourier transform, the Hilbert Huang transform (HHT) offers significant advantages in dealing with non-linear and non-stationary signals^20^. Currently, this method has found extensive applications in the areas of bridge structure damage identification and detection^21^, marine engineering^22^, and earthquake landslide damage identification^23,24^. Zhu et al.^25^, Wu et al.^26^, and Gong et al.^27^ employed the HHT method to process seismic signals, enabling the extraction of the energy time–frequency distribution of various seismic signals. This quantified the significant role of the HHT method in structural analysis and structural control. Wang et al.^28^ and Qin et al.^29^ applied the HHT method to process dynamic monitoring data of bridges, enabling a more accurate and effective identification of modal parameters for large bridge structures. Chen et al.^30^ examined and validated the condition of railroad piers under normal operation and settlement by employing empirical mode decomposition (EMD) and Hilbert–Huang Transform (HHT) spectral analysis theory. The findings revealed the occurrence of settlement during the construction of new piers. In general, the Hilbert–Huang Transform is employed to process non-smooth response signals, offering improved adaptability and various advantages. Meanwhile, the structural response law and vibration wave transmission mechanism of a rotating bridge under train loading of an existing line analyzed from the energy point of view have to be further discussed.

Therefore, this study takes the actual project as an example, through the field test and numerical analysis of Shizuishan Special Bridge of Baoyin Railway. The spatial response characteristics of rotating bridge structures are systematically revealed, and a method of combining the time-domain spectrum with the Hilbert–Huang transform (HHT) energy spectrum is proposed. The peak spectral characteristics and the variation curves of the HHT Peak Marginal Spectral Amplitude (PMSA) at each measurement point are synthesized to elucidate the transmission mechanism of vibration waves in the rotating bridge structure. The comparison results show that the marginal spectrum is superior to the Fourier transform time spectrum, which can accurately reflect the actual frequency components of the vibration signals at each measurement point of the rotating bridge.

## Time–frequency and HHT energy spectrum analysis methods

### The basic principle and characteristics of HHT method

The Hilbert–Huang Transform (HHT) stands out as an adaptive time–frequency analysis technique tailored for nonlinear and non-stationary signals, as introduced by Huang et al.^31^. Rooted in the Hilbert transform, this approach incorporates the Empirical Mode Decomposition (EMD) technique to extricate Intrinsic Mode Functions (IMFs). Diverging from conventional methods, HHT successfully surmounts the constraints of the uncertainty principle, placing emphasis on capturing local signal characteristics. Remarkably, it dispenses with the necessity for a complete time-domain waveform in local frequency analysis while maintaining unwavering frequency resolution throughout. In contrast to exclusively time-domain or frequency-domain analysis methods, HHT provides a transparent representation of the dynamic interplay between spectrum and time, yielding significant strides in signal analysis. The indispensable preprocessing step of HHT involves the Empirical Mode Decomposition (EMD), acting as the foundational signal decomposition method^32^. Through the application of EMD, intricate signals can be deconstructed into a series of IMFs, subsequently subjected to the Hilbert transform for further analysis.

Initially, the complex non-smooth signal is decomposed into a linear superposition of multiple narrowband Intrinsic Mode Functions (IMFs), facilitating the determination of the instantaneous frequency. For the acceleration time series signal *X*(*t*) obtained from the field test, the EMD operation is applied to derive the corresponding IMF components, denoted as *Y*(*t*):

1 $$Y(t) = \frac{1}{\pi }P\int_{ - \infty }^{ + \infty } {\frac{{X(t^{\prime})}}{{t - t^{\prime}}}} dt^{\prime}$$

Note: *P* is the Corsi principal value component value.

Through the acquisition of the eigenmode parameter (IMF) component *Y*(*t*), the construction of the analytic signal *Z*(*t*) becomes feasible, allowing for the derivation of the instantaneous frequency profile of the IMF using a specific analytic equation. The derivation process unfolds as follows:

2 $$Z(t) = X(t) + iY(t) = a(t)e^{i\theta (t)}$$

where: *a*(*t*) is the instantaneous amplitude and $\theta (t)$ is the instantaneous phase, which is expressed as:

3 $$a(t) = \left[ {X^{2} (t) + Y^{2} (t)} \right]^{1/2}$$

4 $$\theta (t) = \arctan \left[ {\frac{X(t)}{{Y(t)}}} \right]$$

In the Hilbert transform, the instantaneous frequency *w*(*t*)is defined as:

5 $$w(t) = \frac{d\theta (t)}{{dt}}$$

Substituting Eq. (3)–(5) into Eq. (2) yields:

6 $$Z(t) = \sum\limits_{j = 1}^{n} {a_{j} } (t,w_{j} )e^{{i\int {w_{j} (t)dt} }}$$

where: $a_{j} (t,w_{j} )$ is the instantaneous amplitude of the *j*th order IMF at moment *t* corresponding to frequency *j*.

The HHT spectrum, denoted as *H*(*t*,*w*), is obtained by combining all the ${\text{a}}_{\text{i}}\text{(}{\text{t}}\text{, }{\text{w}}_{\text{j}}\text{)}$ components and represents the time–frequency distribution of the entire signal amplitude (energy). Its mathematical expression is as follows:

7 $$H(t,w) = \sum\limits_{j = 1}^{n} {a_{j} (t, \, w_{j} )}$$

The expression of HHT marginal spectrum *h* (*w*), *h* (*w*) can be obtained by integrating *H* (*t,w*) in the time domain:

8 $$h(w) = \int_{0}^{T} {H(w, \, t)} dt$$

Through signal analysis in MATLAB 2018a software, the Hilbert energy amplitude of the vibrational wave is adept at accurately and graphically examining the spatial response characteristics of a rotating bridge structure. This analysis is conducted through a three-dimensional exploration of time–frequency–energy. The marginal spectra offer a quantitative depiction of the vibration signal energy distribution across the frequency axis. By scrutinizing the dominant frequency in the marginal spectrum, the transmission mechanism of vibration waves within the rotating bridge structure can be clearly elucidated.

### Construction of time–frequency and HHT energy spectrum analysis methods

**Figure 1:**
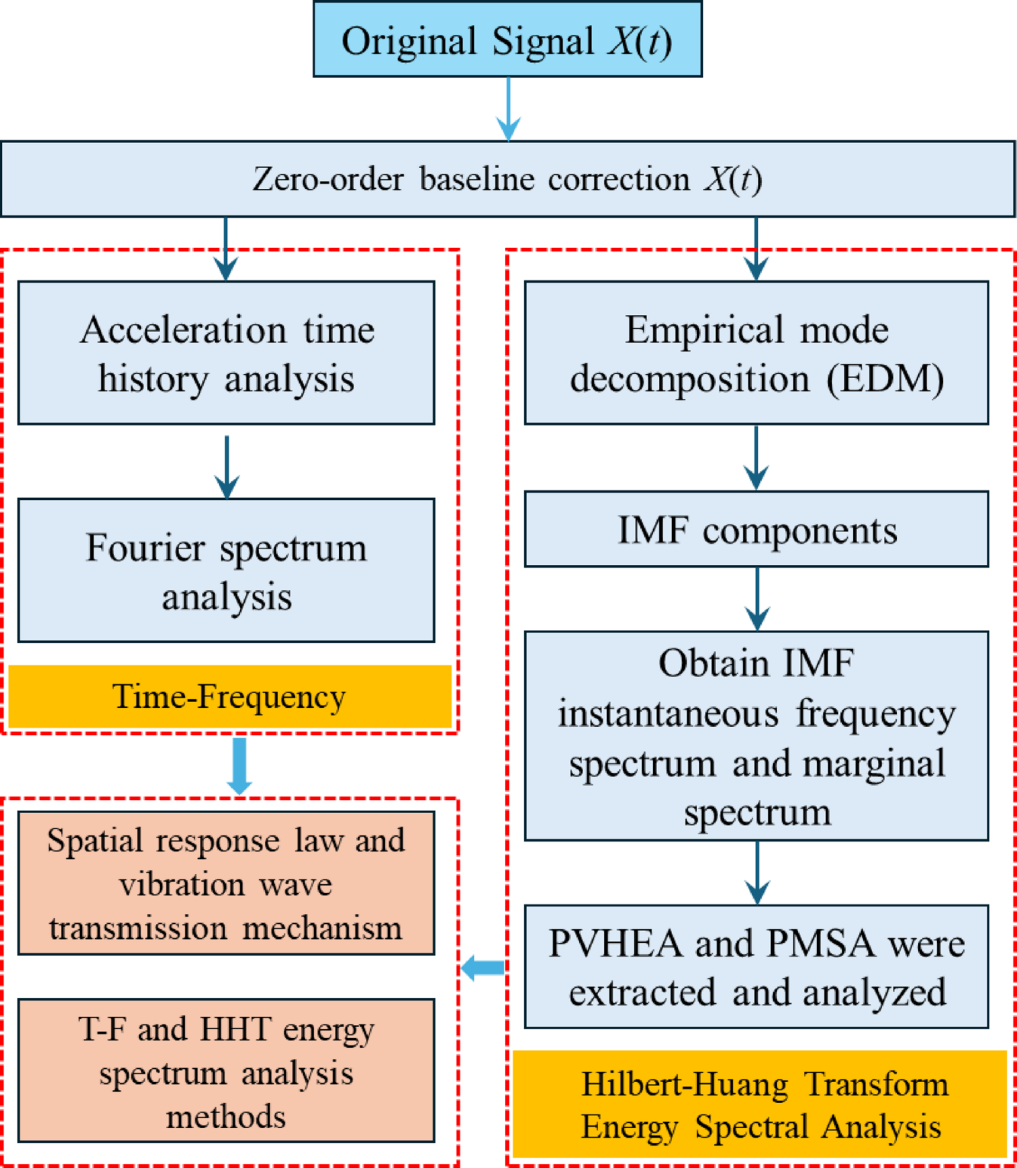
Analytical steps for time–frequency and HHT energy spectral analysis methods.

This article conducts time–frequency analysis and Hilbert–Huang Transform (HHT) energy spectrum analysis on the original signals separately. By comprehensively comparing the results of the two analyses, the system reveals the spatial response law and vibration wave transmission mechanism of the rotating bridge structure under the action of the train load of the existing line. Additionally, the applicability of time–frequency analysis and HHT energy spectrum analysis methods in analyzing the response mechanisms of rotating bridge structures are investigated. The specific analysis steps of the adopted time–frequency and HHT energy spectral analysis methods are illustrated in Fig. 1.

## Field test experiment and modal analysis of rotating bridge

### Purpose of the test

In this experiment, vibration sensors are used to collect field data of the rotating bridge to study the spatial response law and vibration wave transmission mechanism of the rotating bridge structure under the action of the train load of the existing line. The purpose of this paper is to explore the response state of the rotating bridge under different types and speeds of train loads, and to study the applicability of time course spectrum and HHT energy spectrum analysis methods in analyzing the response mechanism of the rotating bridge structure.

### Actual project overview

**Figure 2:**
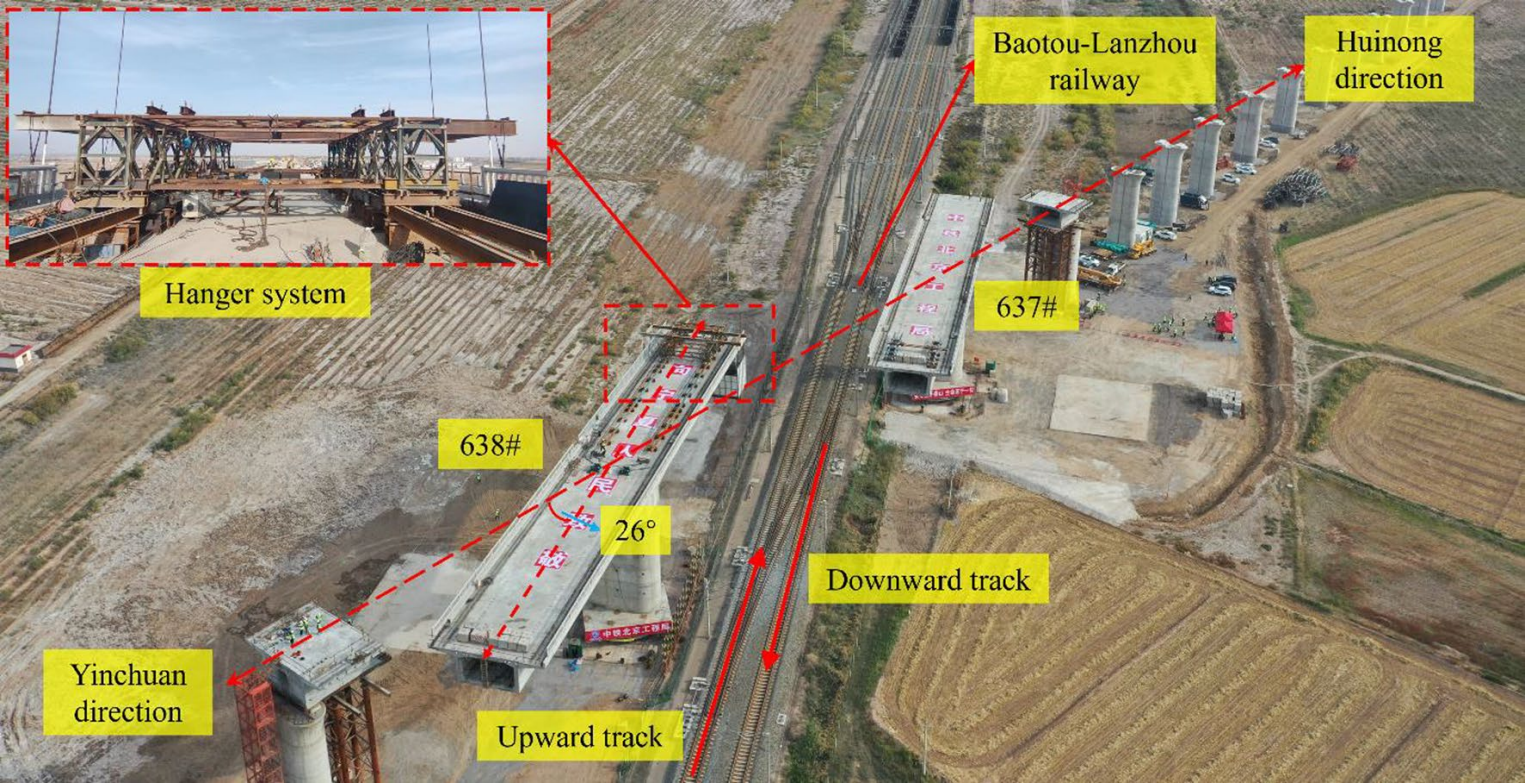
Construction site drawing of (48 + 80 + 48) m swivel of Shizuishan Bridge.

This study is carried out on the rotating continuous beam of the Shizuishan Bridge, which spans a length of (48 + 80 + 48) m on the Huiyin section of the Baotou-Yinchuan Railway. The Baolan Railway is a major trunk line in the western region of China, with a daily traffic volume of more than 40 trains. In order to ensure the uninterrupted operation of the Baotou-Lanzhou Railway, advanced domestic construction techniques for rotating bridges will be adopted, using the “build first and then turn” method. During the construction phase, the bridge will initially run parallel to the existing line and the hanging basket method will be used. Once the cantilever construction is completed, the bridge undergoes a 26° clockwise rotation, enabling the closure construction of the box girders on both sides. The single beam has a rotation radius of 39 m and maintains a clearance of 16 m above the track surface. The total weight of the two T-shaped continuous beams is determined to be 10,400 tons based on weighing test measurements, and a 6000-ton reactive powder concrete spherical hinge is designed and selected. The primary study was on side 638#, which has a minimum distance of 1.5 m from the railway fence. The main pier foundation is supported by 18 bored piles measuring 90 m in length and 1.5 m in diameter. The scene diagram of the Shizuishan Bridge (48 + 80 + 48) m before the swivel is shown in Fig. 2.

### Field test design and sensor arrangement

**Figure 3:**
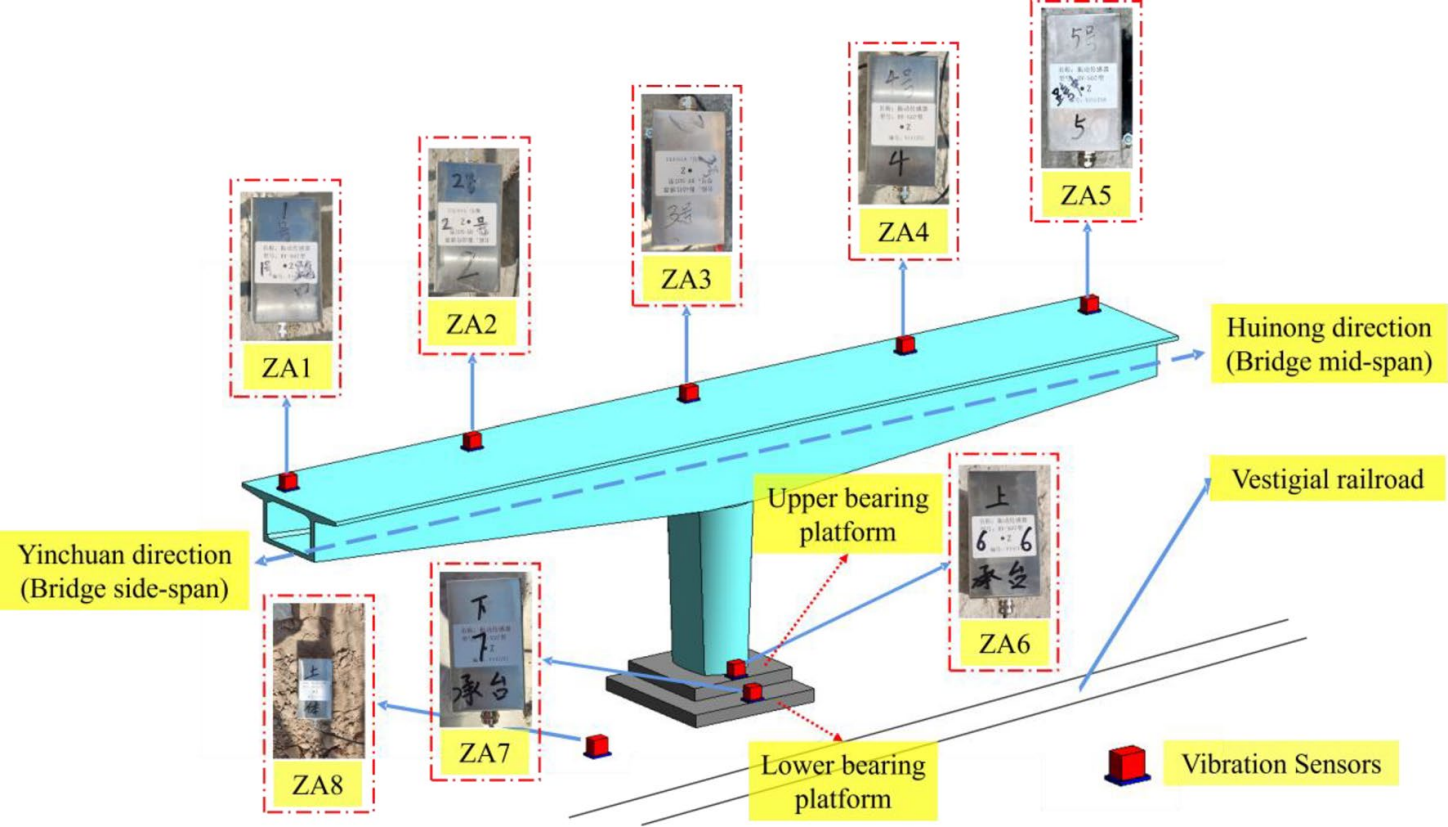
Schematic diagram of field test sensor placement.

In this experiment, vertical vibration sensors were installed at the construction site of the rotating bridge to dynamically collect the vertical acceleration response of the bridge. The experimental setup incorporated the BY-S07 low-frequency dynamic coil reciprocating vibration sensor. This sensor utilizes advanced passive closed-loop servo feedback technology and exhibits excellent low-frequency characteristics and impedance matching properties, enabling precise acquisition of low-frequency (down to 0.14 Hz) and large displacement (500 mm) vibration signals. In order to study the spatial response law and vibration wave transmission mechanism of the rotating bridge structure under the action of the train load of the existing line, a total of 8 BY-S07 sensors were deployed. These sensors were placed at specific locations, including the side-span (ZA1), half side-span (ZA2), center of block 0# (ZA3), half mid-span (ZA4), mid-span (ZA5), upper bearing pedestal (ZA6), lower bearing pedestal (ZA7), and the soil near the foundation of the rotating bridge (ZA8) on the 638# side of the rotating bridge. For the exact placement locations, please refer to Fig. 3.

**Figure 4:**
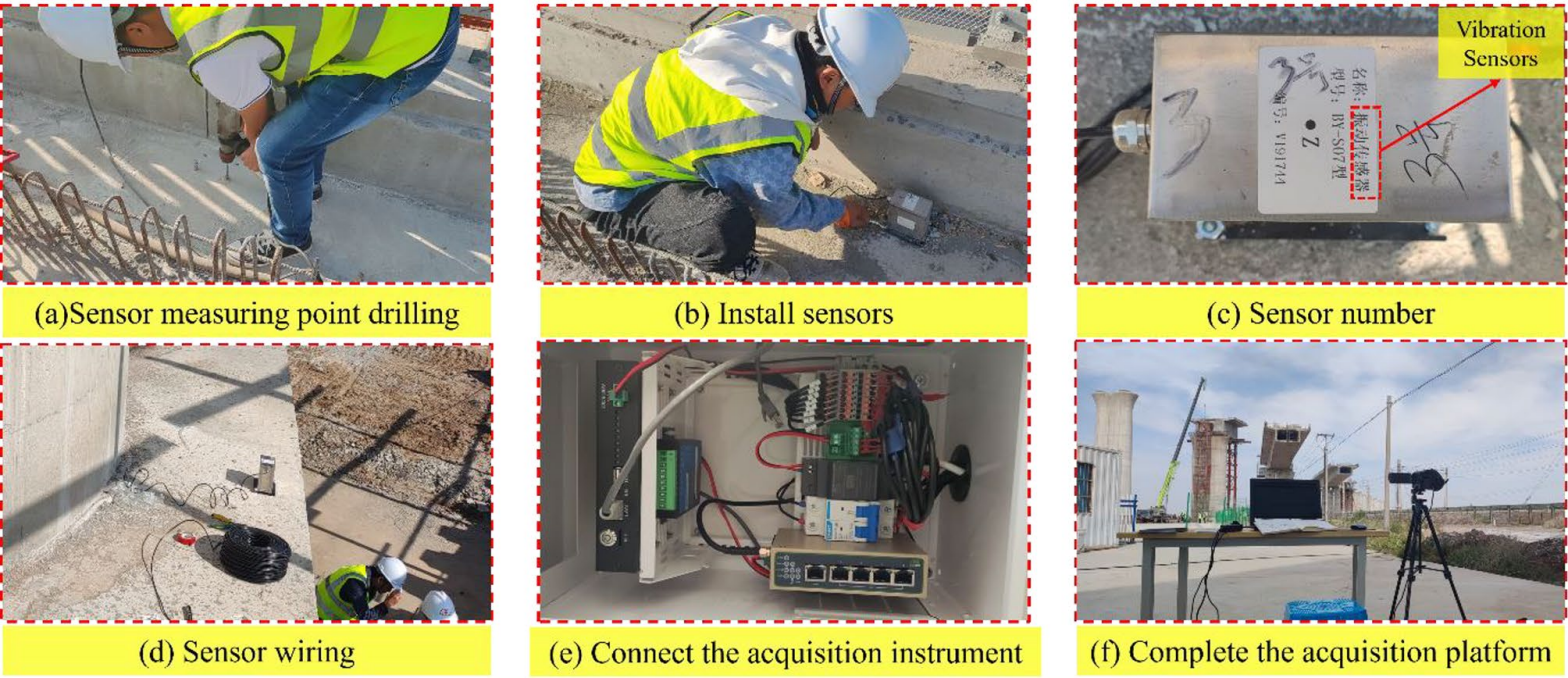
Field test vibration sensor monitoring system deployment process.

Installation and deployment of the test site was carried out in accordance with the test design plan. In order to avoid any disruption to the normal construction progress of the rotating bridge, the outside of the bridge deck was chosen for the installation of the vibration sensors. The specific locations of these sensors were determined by measurements taken at the test site. An electric percussion drill was used to drill holes at the designated measurement points. After drilling, a blower was used to remove debris from the holes and expansion bolts were inserted. The vertical vibration sensors were then securely attached to their respective measurement points, ensuring that each sensor was pointing upwards to facilitate accurate data collection. Each vibration sensor was then given a unique number, marked accordingly and the wiring connected to the collector. The configuration of the cloud-based platform was then completed and a data acquisition test was performed. Figure 4 illustrates the specific preparation process for the field test.

### Test program

**Figure 5:**
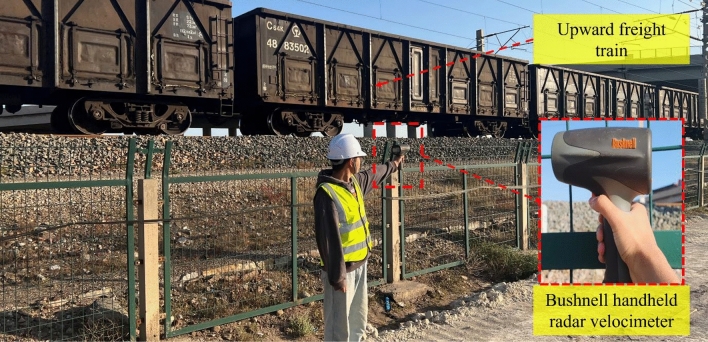
On-site speed measurement of existing lines.

To ensure the reliability of the field tests, the data collection was conducted when no related construction operations were being carried out on the rotating bridges. During the field experiment, a handheld radar speedometer manufactured by Bushnell Company in the United States was used to collect and record the speed of the passing trains. The situation of speed measurement on site is shown in Fig. 5. To investigate the response state of the rotating bridge under various working conditions, different types of trains at different speeds were selected as research conditions. The selected trains in this study are primarily categorized into two types: passenger trains and freight trains. Specifically, the freight train is the C64 open wagon, organized in a 45-car formation, with a rated axle load of 22.2 t and a fixed distance of 8.7 m. The passenger train is the 25T passenger car, organized in a 15-car formation, with a rated axle load of 15.5 t and a fixed distance of 18 m. The selected trains are all “Upward track-trains”. For specific working conditions, please refer to Table 1.

**Table 1:** Test loading condition for trains.

Case	Train types	Direction of train	Train speed *v* (km/h)	Load capacity
1	Freight train	Upward track	101	Full load
2	Freight train	Upward track	71	Full load
3	Passenger train	Upward track	104	–
4	Passenger train	Upward track	74	–

### Three-dimensional finite element model of rotating bridge and its modal analysis

**Figure 6:**
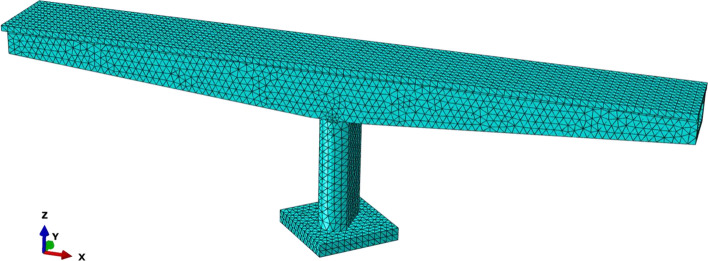
Mesh model used in the calculations.

The modal parameters of the bridge structure reflect its own characteristics and are an important assessment basis for structural resonance analysis. Therefore, according to the relevant physical parameters of the engineering prototype of the rotating bridge, the modal parameters of the rotating bridge are analyzed based on the finite element analysis software ABAQUS. In the finite element model, the main girder is a variable cross-section continuous box girder, the material is C50 concrete, the material of the pier and bearing platform is C35 concrete, and the pier and girder are temporarily cemented. Based on the linear regression analysis step in the implicit solution function of the software, the modal parameters of the rotating bridge are analyzed. Specifically, the Lanczos solver is used to numerically calculate the modal parameters of the rotating bridge. The mesh model used for the whole bridge is shown in Fig. 6.

**Figure 7:**
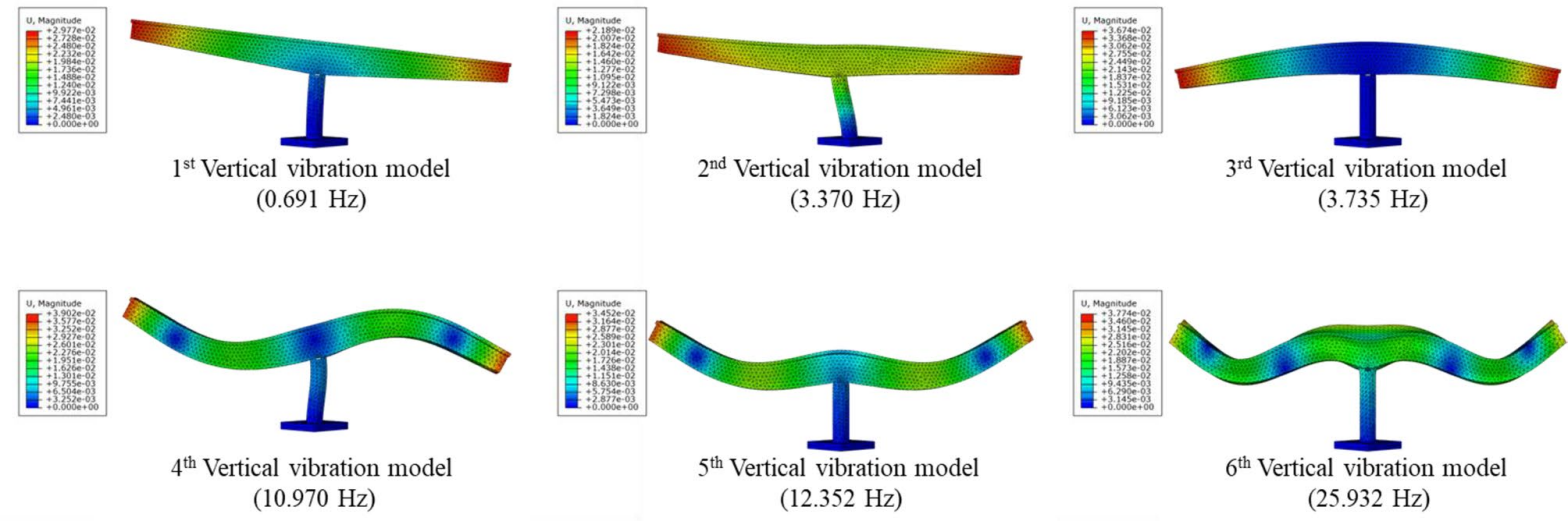
The first six order vertical vibration mode diagram of rotating bridge.

Based on the modal analysis in numerical simulation, the self-oscillation characteristics of the rotating bridge can be obtained and the dynamic deformation characteristics of the bridge can be identified. Since this study mainly focuses on analyzing the vertical vibration response characteristics of the rotating bridge. Therefore, the first six vertical intrinsic vibration modes and intrinsic frequencies of the rotating bridge are obtained through modal analysis, as shown in Fig. 7. According to Fig. 7, the inherent frequency of vertical vibration of the rotating bridge is mainly distributed within 20 Hz. Therefore, the focus should be on the 2–5 Hz and 10–13 Hz frequency bands in the subsequent study of the vertical response of the rotating bridge structure under the dynamic loading of the train on the existing line.

## Analysis of experimental results

### Time–frequency spectrum response analysis of rotating bridge under train load on existing railway line

To overcome the problem of zero drift in the vibration acceleration data collected, a zero order baseline correction operation is performed. This operation consists of the following steps: First, the acceleration data from each measurement point is collected during the period when no train is passing under environmental excitation, and an averaging operation is performed. Subsequently, the acceleration data from each measurement point under different working conditions is subtracted from the calculated average value to obtain the zero-order baseline correction.

**Figure 8:**
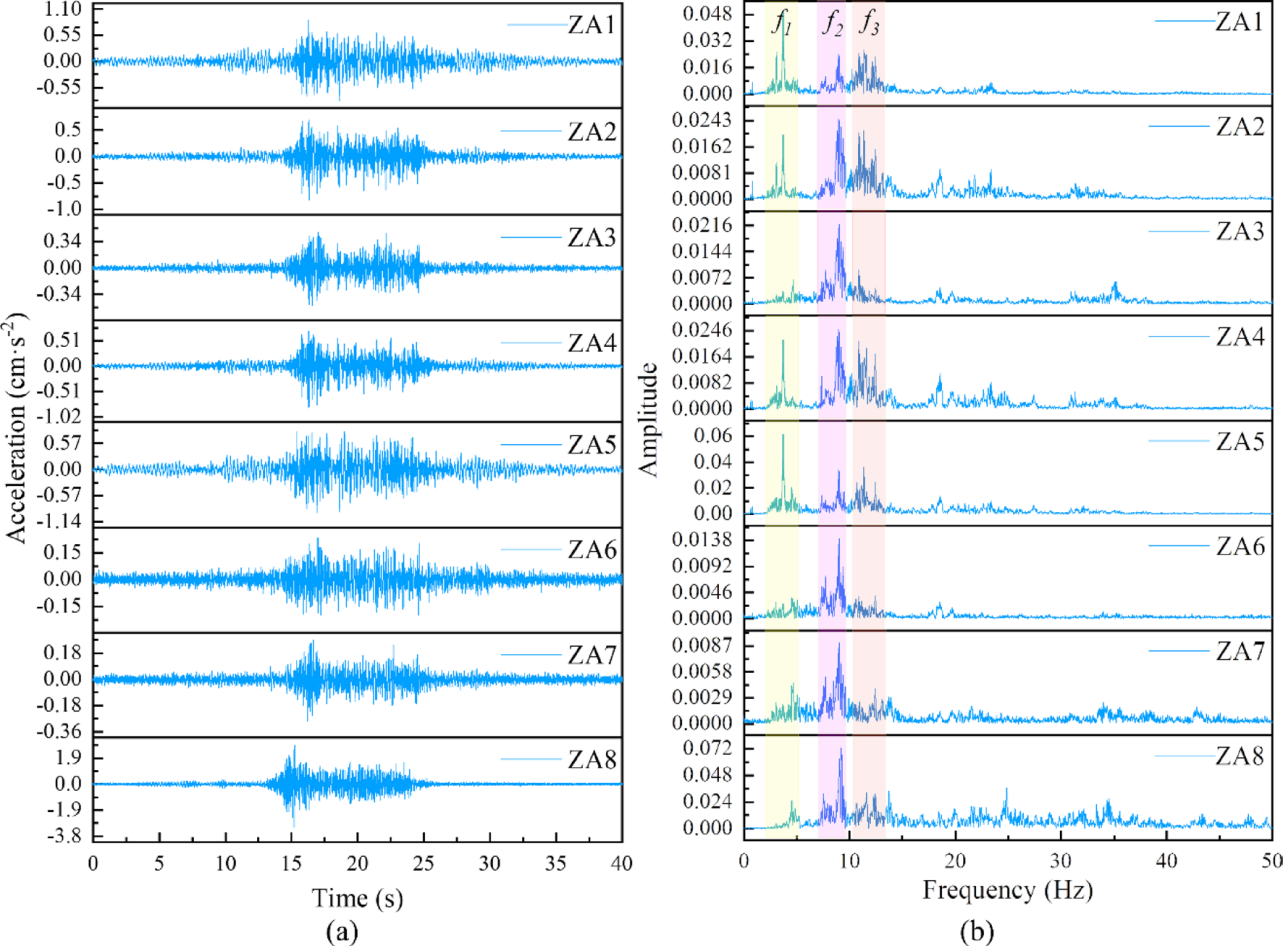
The measuring points when a fully loaded freight train is traveling in the upward direction: (**a**) acceleration-time curve; (**b**) Fourier spectrum.

In order to study the spatial response law and the vibration wave transmission mechanism of the rotating bridge structure under the influence of the train load of the existing line, we specifically chose an up-track freight train with a speed of 101 km/h as a research object. Acceleration amplitude-time curves and corresponding Fourier spectra were obtained for each measurement point on the rotating bridge. The specific results are shown in Fig. 8. From Fig. 8, under the influence of train loads on the existing lines, the measurement point (ZA8) located near the foundation of the revolving bridge has the highest acceleration response amplitude. In contrast, the acceleration at the measurement point located at the lower bearing platform of the swing bridge (ZA7) is significantly reduced, with a peak value of approximately 1/10 of the former. This observation suggests that the interaction between the deep soil layers in the foundation of the rotating bridge affects the acceleration response, and the stiffness of the foundation may limit the dynamic response of the rotating bridge. In addition, the soil between the existing track and the foundation of the rotating bridge has favorable energy dissipation characteristics. Under the effect of the train load, the friction and energy dissipation between the particles in the soil convert the energy of the existing train load into the internal strain energy, thus greatly reducing the vibration signal transmitted to the rotating bridge structure.

As the vibration wave propagates, there is a certain attenuation in the peak acceleration at the measurement point on the upper bearing platform (ZA6). This attenuation occurs due to the absorption of energy during the transmission of the vibration wave through the spherical hinge of the rotating bridge. Subsequently, the vibration wave passes through the pier structure from the upper bearing pedestal (ZA6) to the beam body of the rotating bridge. The acceleration peak is amplified at the center of the 0# block (ZA3) in the beam body. From there, the vibration wave propagates to the ends of the beam, reaching the side span (ZA1) and the center span (ZA5). Throughout this process, the acceleration undergoes significant amplification and exhibits a symmetrical distribution pattern during transmission.

Based on the results of the modal analysis of the rotating bridge in the previous paper and combined with the peak distribution law of the Fourier spectrum. Three main frequency bands are selected for analysis in Fig. 8, namely: *f*_1_ (2–5 Hz), *f*_2_ (7–9 Hz) and *f*_3_ (10–13 Hz). The peak statistics of the extracted *f*_1_ and *f*_*3*_ bands at different measurement points are shown in Table 2. From Table 2, the components in the frequency range of *f*_1_ (2–5 Hz) and *f*_3_ (10–13 Hz) show significant amplification during the transmission of the vibration wave through the center position of block 0# of the beam to the beam end of the beam. This indicates that *f*_1_ and *f*_3_ are the natural frequencies (vertical frequencies of the bridge) of the rotating bridge structure, while the frequency band *f*_2_ (7–9 Hz) corresponds to the frequency components induced by the loading of existing railway trains. In summary, based on the analysis of the spectrum data, it can be concluded that the low-frequency component *f*_1_ is the primary factor contributing to the amplification of the acceleration response in the rotating bridge, while *f*_3_ plays a secondary role. These findings are highly significant for enhancing our understanding of the vibration response mechanism in rotating bridges.

**Table 2:** Peak value table of each frequency band at different measuring points.

Measuring point	The peak of *f*_1_ frequency band	Magnification times	The peak of *f*_3_ frequency band	Magnification times
ZA1	0.0494	7.60	0.0265	2.82
ZA2	0.0200	3.08	0.0212	2.26
ZA3	0.0065	–	0.0094	–
ZA4	0.0217	3.34	0.0213	2.27
ZA5	0.0614	9.45	0.0362	3.85

**Figure 9:**
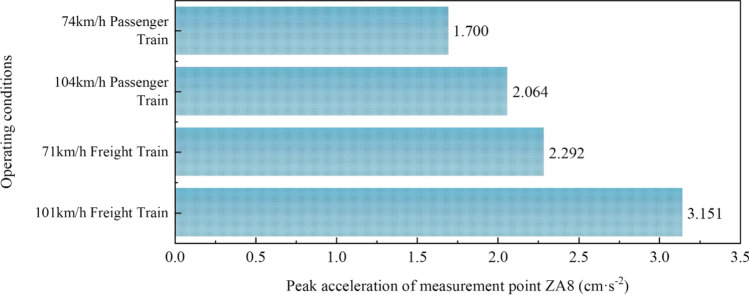
Peak acceleration at ZA8 measurement point under different train load conditions.

**Figure 10:**
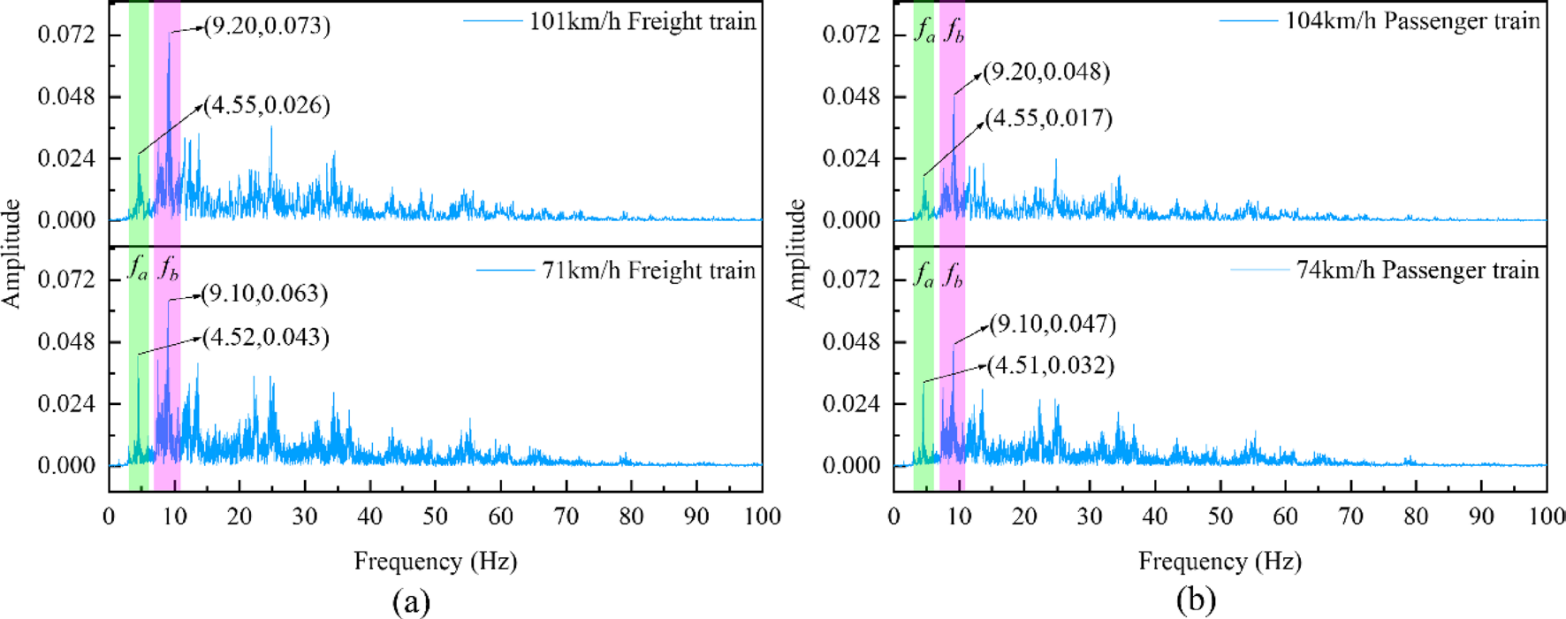
ZA8 measurement point acceleration frequency spectrum curves: (**a**) Freight train; (**b**) Passenger train.

To investigate the impact of various types and speeds of train loads on the soil near the foundation of the rotating bridge, peak acceleration graphs were generated for each measurement point of the girders under various working conditions. The curves displayed in Fig. 9 illustrates the peak acceleration of the soil (ZA8) near the foundation of the rotating bridge under various working conditions. Additionally, Fourier transform (FFT) was applied to the acceleration signals from the measurement point near the foundation of the existing line rotating bridge (ZA8) under diverse working conditions, yielding the corresponding Fourier spectra. The specific results of the Fourier spectra are depicted in Fig. 10.

According to Fig. 9, it is observed that the soil vibrations induced by the dynamic load of existing railway trains exhibit the following patterns: for trains of the same type, higher-speed trains result in a larger acceleration response; for trains at the same speed, freight trains induce a greater acceleration response.

The measuring point near the foundation of the existing line rotating bridge, referred to as ZA8, serves as the sole reference point for the input excitation of the rotating bridge. By analyzing this measurement point, the response characteristics of the input excitation of the rotating bridge system can be revealed. Therefore, from Fig. 8, it can be observed that the frequency bands of the influence of the existing line train loads on the surrounding soil body mainly range from 0–80 Hz. However, within the range of 11–80 Hz, there are multiple peaks coexisting, making the analysis more challenging. Considering this, the present study primarily focuses on researching the *f*_a_ (3–6 Hz) and *f*_b_ (7–11 Hz) typical frequency bands. Under the action of the same type of train, the spectrum of the vibration wave generated is varied by the speed of the train. According to the research of related scholars^33–35^, under the condition that the fixed distance of the train pair is fixed, with the increase of the train speed, the frequency spectrum of the generated vibration shows an increase in the high-frequency band and a weakening of the low-frequency band. By analyzing the Fourier spectrum data of the soil near the foundation of the existing railway rotating bridge (ZA8), with particular attention to the *f*_a_ and *f*_b_ frequency bands, it can be observed that the amplitude of the *f*_b_ frequency band increases with the speed of the existing railway train, while the amplitude of the *f*_a_ frequency band decreases.

The analysis has shown that the peak ratios of the *f*_*b*_ and *f*_*a*_ bands for the 101 km/h freight train and the 104 km/h passenger train are consistently maintained at around 2.82(*f*_*bF*_/*f*_*aF*_ = *f*_*bP*_/*f*_*aP*_ = 2.82). Similarly, the peak ratios of the *f*_b_ and *f*_a_ bands for the 71 km/h freight and 74 km/h passenger trains are consistently around 1.47(*f*_*bF*_/*f*_*aF*_ = *f*_*bP*_/*f*_*aP*_ = 1.47). These results confirm that as the train speed increases, the frequency spectrum of the induced vibration wave shifts from the low frequency component *f*_a_ to the high frequency component *f*_b_. The results show that train speed has a significant effect on the frequency distribution characteristics of the vibration wave. In scenarios where speeds are relatively similar, the amplitude of each frequency band in the Fourier spectrum of passenger trains is comparatively small. In addition, the peak ratios of the corresponding frequency bands remain consistently similar across different trains. The ratio of the peak between the *f*_a_ and *f*_b_ bands for a 101 km/h freight train to that of the corresponding frequency band for a 104 km/h passenger train remains consistently fixed at about 1.52(*f*_*aF*_/*f*_*aP*_ = *f*_*bF*_/*f*_*bP*_ = 1.52). Similarly, the ratio of the peak value between the *f*_a_ and *f*_b_ bands for a 71 km/h freight train to that of the corresponding frequency band for a 74 km/h passenger train remains consistently fixed at about 1.34(*f*_*aF*_/*f*_*aP*_ = *f*_*bF*_/*f*_*bP*_ = 1.34). The results indicate that the train type has a minimal influence on the frequency distribution characteristics of the vibration wave and primarily affects the overall amplitude response of the vibration wave.

**Figure 11:**
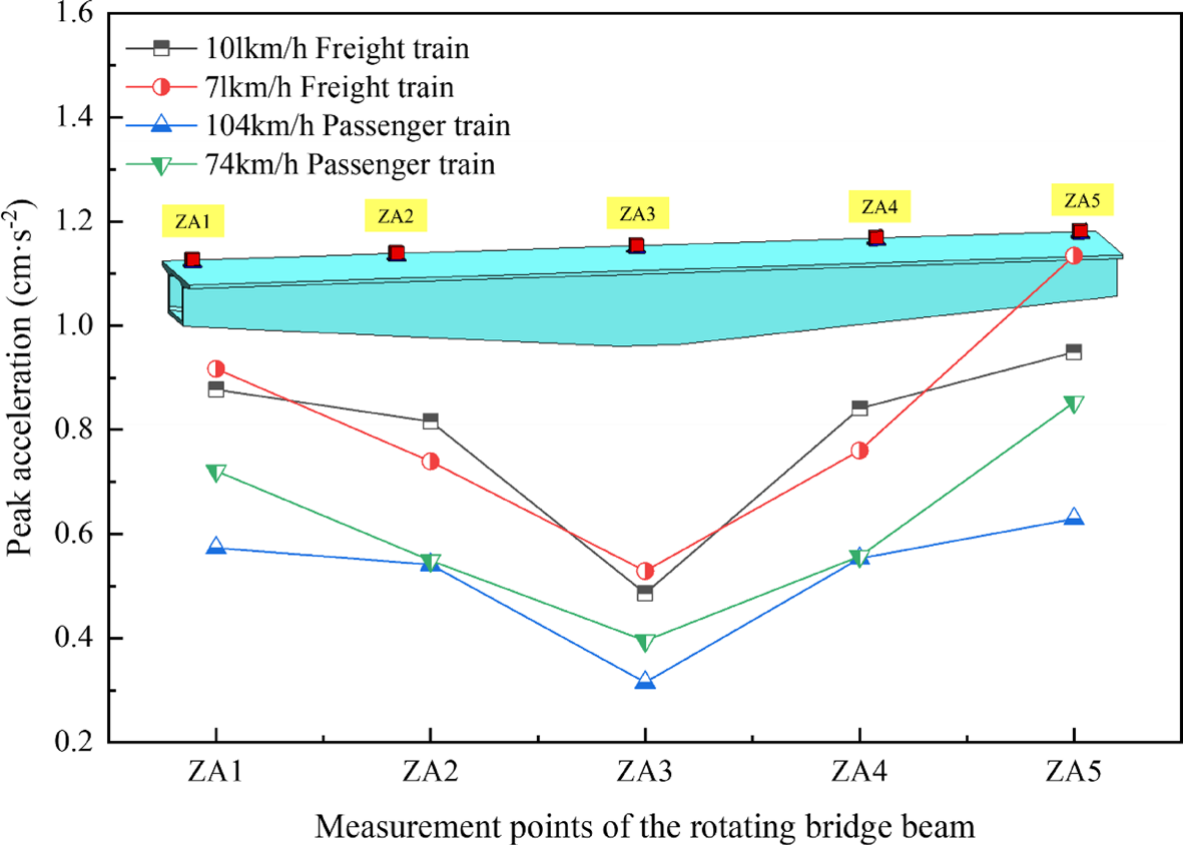
Acceleration peak response curves of each measurement point of the beam under different working conditions.

In order to understand the peak acceleration at each measurement point of the girders of the rotating bridge under various operating conditions, the peak acceleration response curves of the girders at each measurement point were plotted, as shown in Fig. 11.

By analyzing Fig. 11, it is observed that the acceleration peaks of each measuring point on the rotating bridge beam body follow a consistent trend under varying train loads. This trend reveals a gradual increase in peak acceleration from the measurement point of block 0# (ZA3) to the end of the beam, forming an overall “V”-shaped distribution.

Under the same type of train load, the peak acceleration of most measurement points generally decreases with increasing train speed, and this effect is particularly noticeable for passenger trains. In particular, it is worth noting that there is a significant difference between measurement points ZA2 and ZA4, where there is even an occurrence of no change or an increase. In conjunction with the preceding text, as the train speed increases, the peak of the vibration wave generated by the train's dynamic load decreases in the *f*_a_ (3–6 Hz) frequency band and increases in the *f*_b_ (7–11 Hz) frequency band. Due to the superposition of the factors from these two frequency bands, differences appear at measurement points ZA2 and ZA4. When the train speed is similar, the distribution patterns of peak acceleration at each measurement point of the beam caused by different types of train loads are largely the same, except for more pronounced peak acceleration caused by freight trains. Thus, drawing from the preceding section, one can infer that variations in train speed exert a notable influence on the frequency characteristics of excitation vibration waves introduced into the rotating bridge system. The vibration wave generated by the low-speed train on the existing line is more concentrated in the *f*_a_ (3–6 Hz) frequency band. Furthermore, due to the low-frequency component *f*_1_ (2–5 Hz) being the primary factor amplifying the induced rotational bridge acceleration response, coupled with the fact that the *f*_a_ frequency band component of vibrations generated by slow-moving trains overlaps with the 2nd and 3rd vertical natural frequencies of the rotating bridge, resonance occurs. This results in a more pronounced response at the beam-end measurement points under low-speed conditions. Consequently, the frequency distribution characteristics of vibration waves caused by train loads on the existing line have a significant influence on the acceleration response of the rotating bridge's girders.

### Hilbert energy spectrum analysis of vibration waves

**Figure 12:**
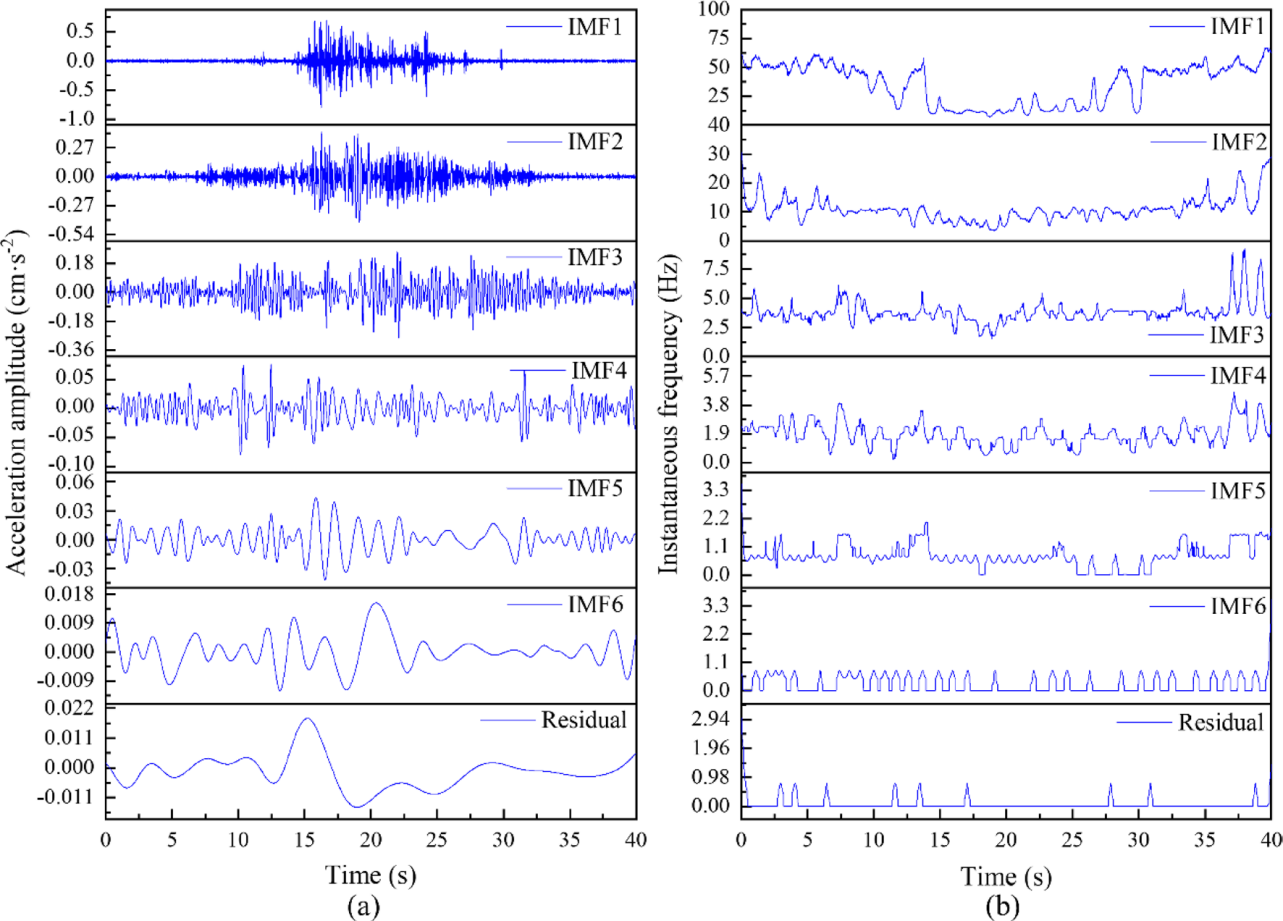
EMD results of the ZA7 measurement point during the upward fully loaded freight train: (**a**) IMF1-Residual; (**b**) the corresponding instantaneous frequencies of the IMFs.

The empirical mode decomposition (EMD) technique is utilized to decompose the acceleration time-history signal obtained from the measured point of the lower bearing platform (ZA7) of the rotating bridge during the operation of the upward full-load freight at a speed of 101 km/h. This decomposition yields corresponding instantaneous spectra, as shown in Fig. 12. The energy spectrum is derived by squaring the instantaneous spectrum of each empirical mode function (IMFs) component. By summing the energy spectra of all IMFs components, the corresponding Hilbert energy spectrum is obtained. By analyzing the Hilbert energy spectrum of the vibration wave, the structural dynamic response characteristics of a rotating bridge under the loading of an existing line train can be revealed in the time–frequency domain.

**Figure 13:**
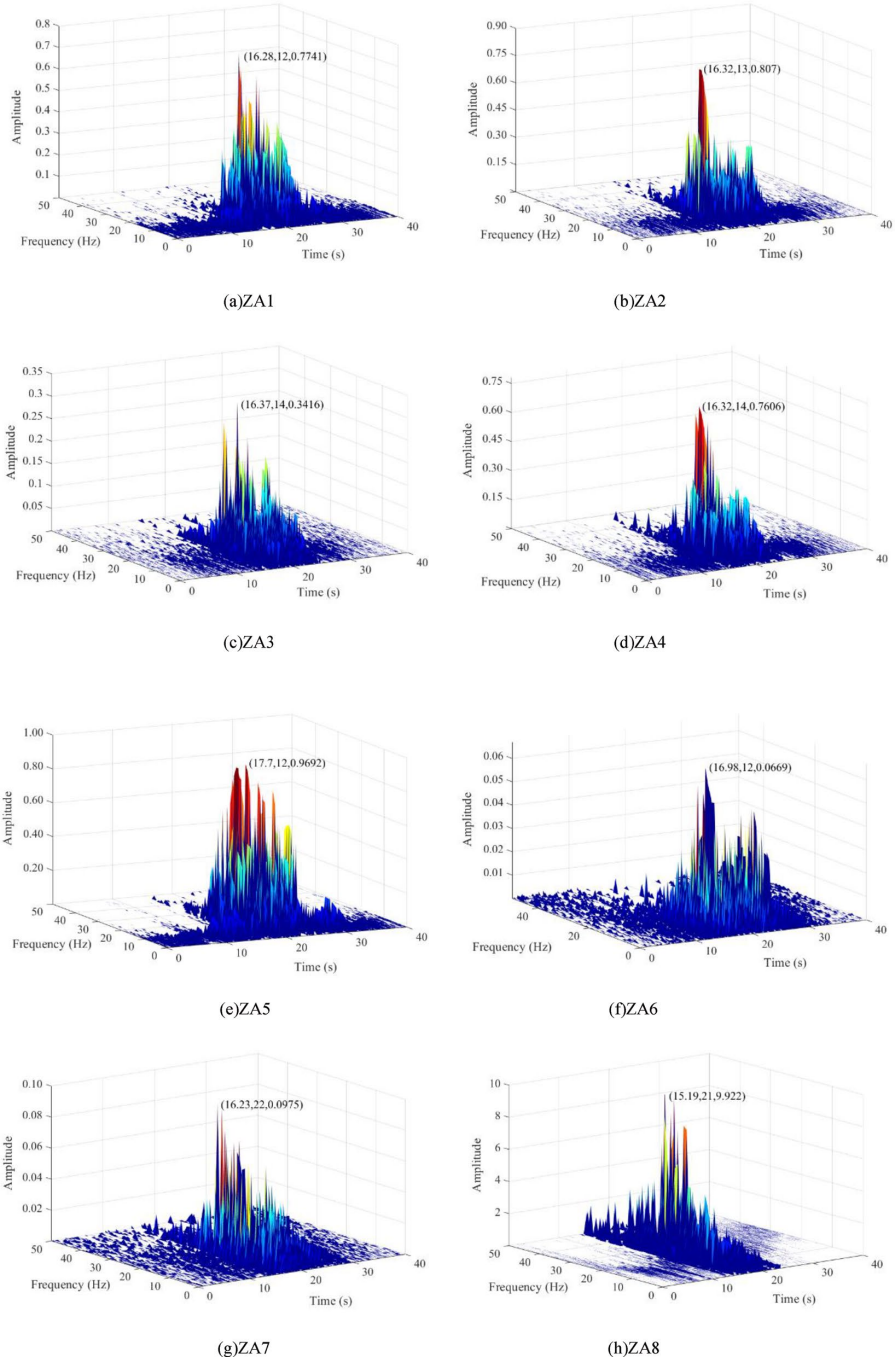
Hilbert energy spectrum of vibration waves at each measurement point of a rotating bridge during operation of an upstream fully loaded freight at 101 km/h.

The Hilbert energy spectra of the vibration waves at different measurement points of the rotating bridge can be obtained by combining the instantaneous spectra of all the empirical modal function (IMFs) components. The Hilbert energy spectra of vibration waves at different measurement points for a 101 km/h full load upward track freight train operation are shown in Fig. 13.

**Figure 14:**
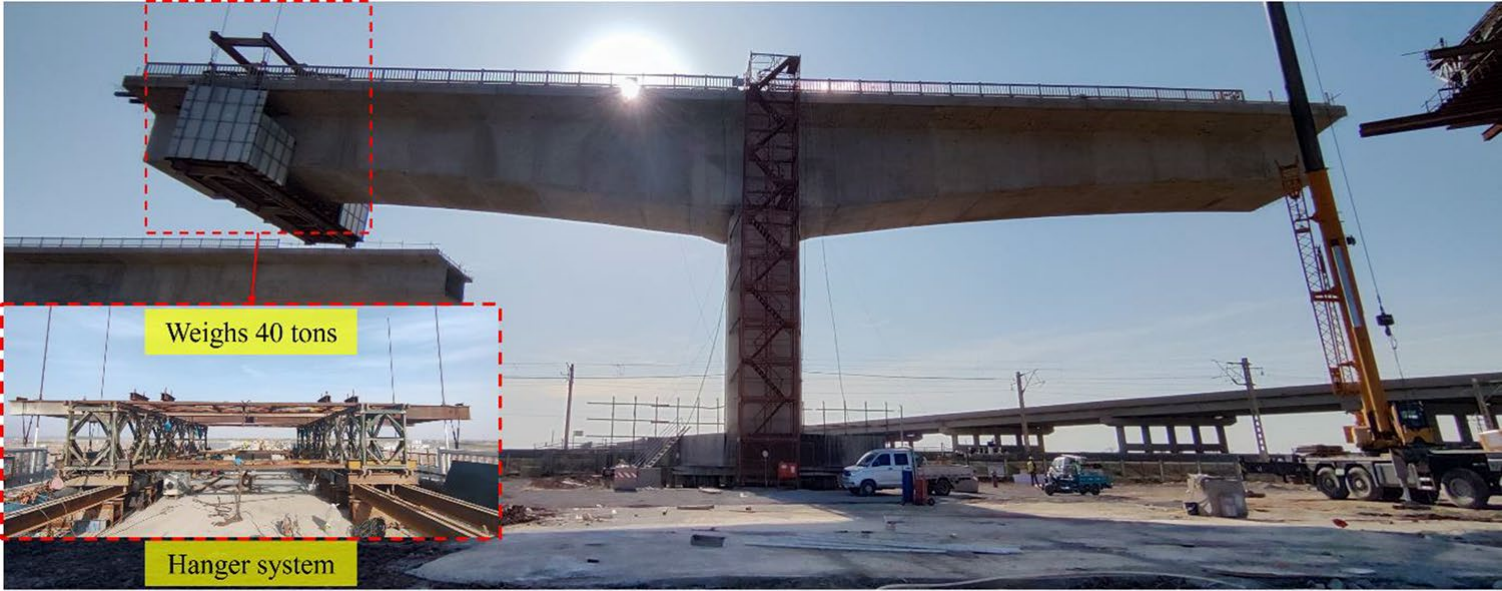
Rotating bridge hanger system.

**Figure 15:**
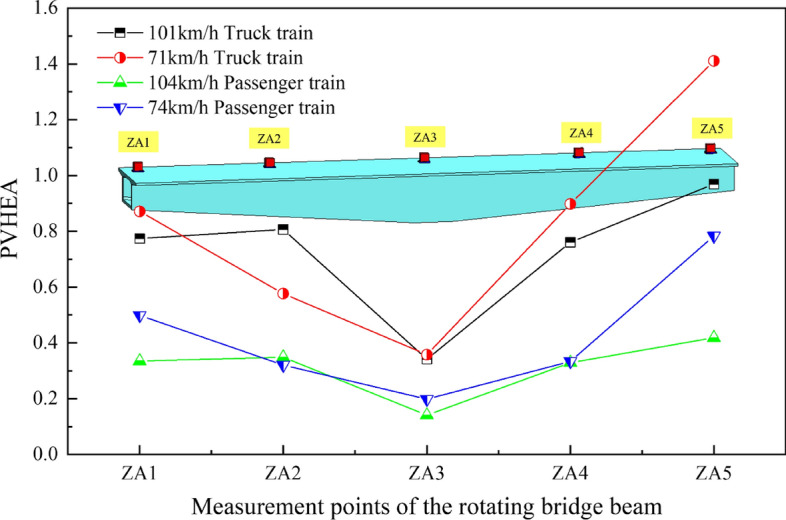
PVHEA of each measurement point of the girders of a rotating bridge under different working conditions.

From Fig. 13, it can be analysed from the three-dimensional perspective of time–frequency–energy at each measurement point that the Peak Vibration-wave Hilbert Energy Amplitude (PVHEA) and shape change significantly for each measurement point of the girders in the rotating bridge under the load of the existing railway line. In particular, the energy shows an increasing trend during the transmission from the measurement point at the 0# block (ZA3) to the end of the girder. Combined with the previous analysis, this situation occurs due to resonance between the vibration wave and the natural frequency of the rotating bridge during the transmission process, resulting in a significant increase in the amplitude of the local frequency band. The PVHEA of each measuring point of the rotating bridge beam is mainly concentrated in the range of 15–25 s in the time domain, and in the frequency domain, it is mainly concentrated in the range of 3–15 Hz. It is worth noting that, similar to the previous analysis, the degree of PVHEA amplification differs significantly between the side span (ZA1) and centre span (ZA5) measuring points. The main reason for this difference is the presence of a mid-span closure hanger system at the mid-span position (Fig. 14), which carries a load of approximately 40 tonnes. The concentrated load results in a relatively high energy concentration at the mid-span position, resulting in a slightly higher PVHEA compared to other positions. Along the transmission path of the vibration wave, the shape of the Hilbert energy spectrum shows a richer frequency component near the peak of the spectrum and a distinct multi-peak phenomenon, indicating the existence of resonance at frequencies near the peak of the spectrum. Based on the above methods, Hilbert energy spectra under different working conditions were drawn respectively, PVHEA of each measuring point of the beam body was extracted under different working conditions, and PVHEA distribution of each measuring point under different working conditions was drawn, as shown in Fig. 15.

After analysing Fig. 15, the spatial distribution of PVHEA of the girders of the rotating bridges under different types and speeds of trains on the existing line shows a consistent pattern, with an overall “V” distribution trend.

Under the condition of the same type of train, the PVHEA of most measuring points is smaller when the train speed is fast. The previous analysis indicates that the frequency distribution characteristics of vibration waves induced by train loads on the existing line significantly influence the response of the rotating bridge girders, with speed playing a major role in determining the frequency distribution characteristics of these waves. Specifically, the vibration waves generated at lower speeds tend to be concentrated in the low frequency component in the same frequency as the *f*_1_ (2–5 Hz) band, resulting in severe resonance of the beam. Under the same speed train load, the PVHEA of the rotating bridge body vibration wave caused by freight train is obviously greater than that caused by passenger train. First, because freight trains are at full load, the load is larger and the vibration is stronger. In addition, the spacing of freight trains is smaller, which leads to more concentrated load effects and more obvious effects on bridge vibration.

In summary, the authors of this study have, for the first time, applied the Hilbert–Huang Transform (HHT) energy spectrum to the investigation of the response of rotating bridge structures. By combining the commonly used time-history spectrum analysis, the response state of the rotating bridge under the influence of train loads on existing lines can be presented from a three-dimensional perspective of time–frequency–energy. This method can deeply interpret the energy situation of each measuring point of the rotating bridge structure in the time and frequency range, and bring a certain role in promoting the stability analysis of the rotating bridge structure. Therefore, the method based on time history spectrum and HHT energy spectrum signal processing and vibration analysis has good applicability in the study of the response mechanism of rotating bridge structure.

### Energy transfer characteristics of vibration waves

**Figure 16:**
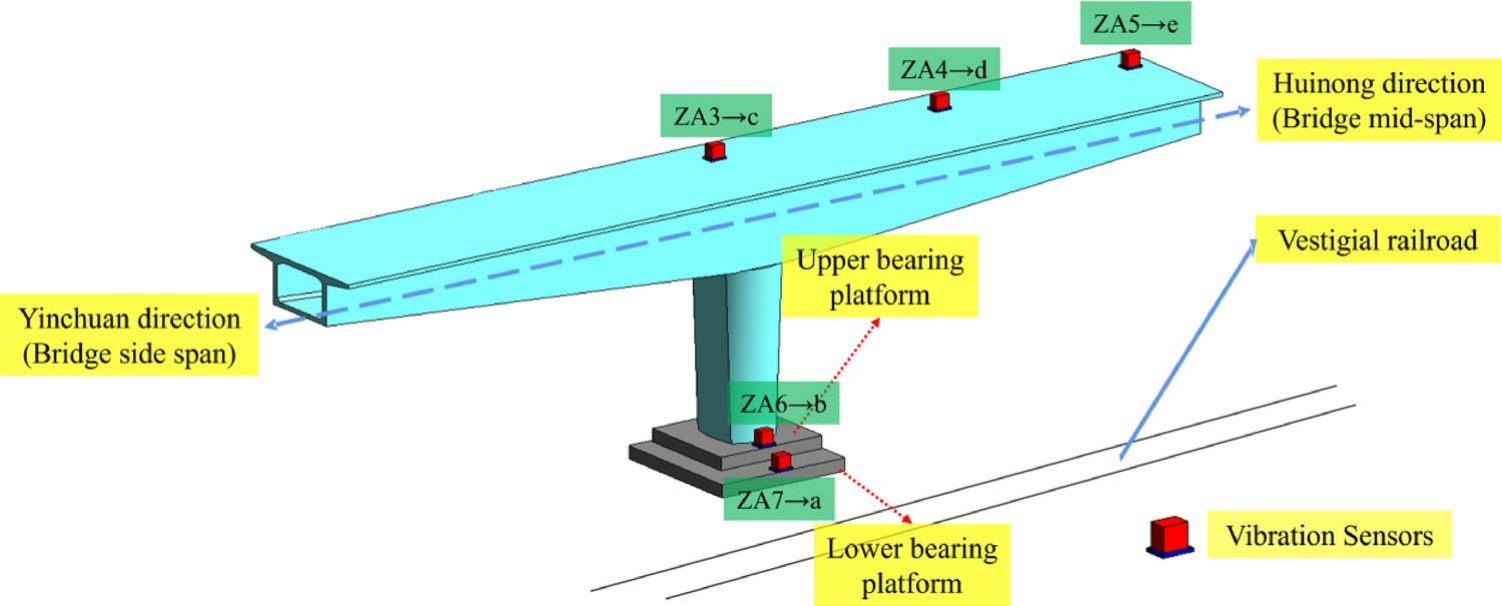
Schematic diagram of partially renumbered measurement points.

To further elucidate the transmission characteristics of the vibration wave energy, based on the previous analysis, it is observed that the acceleration response is more pronounced on one side of the hanging basket. Therefore, the side of the basket was selected for analysis. Meanwhile, for the sake of clarity, some measurement points have been uniformly renumbered, as shown in Fig. 16.

**Figure 17:**
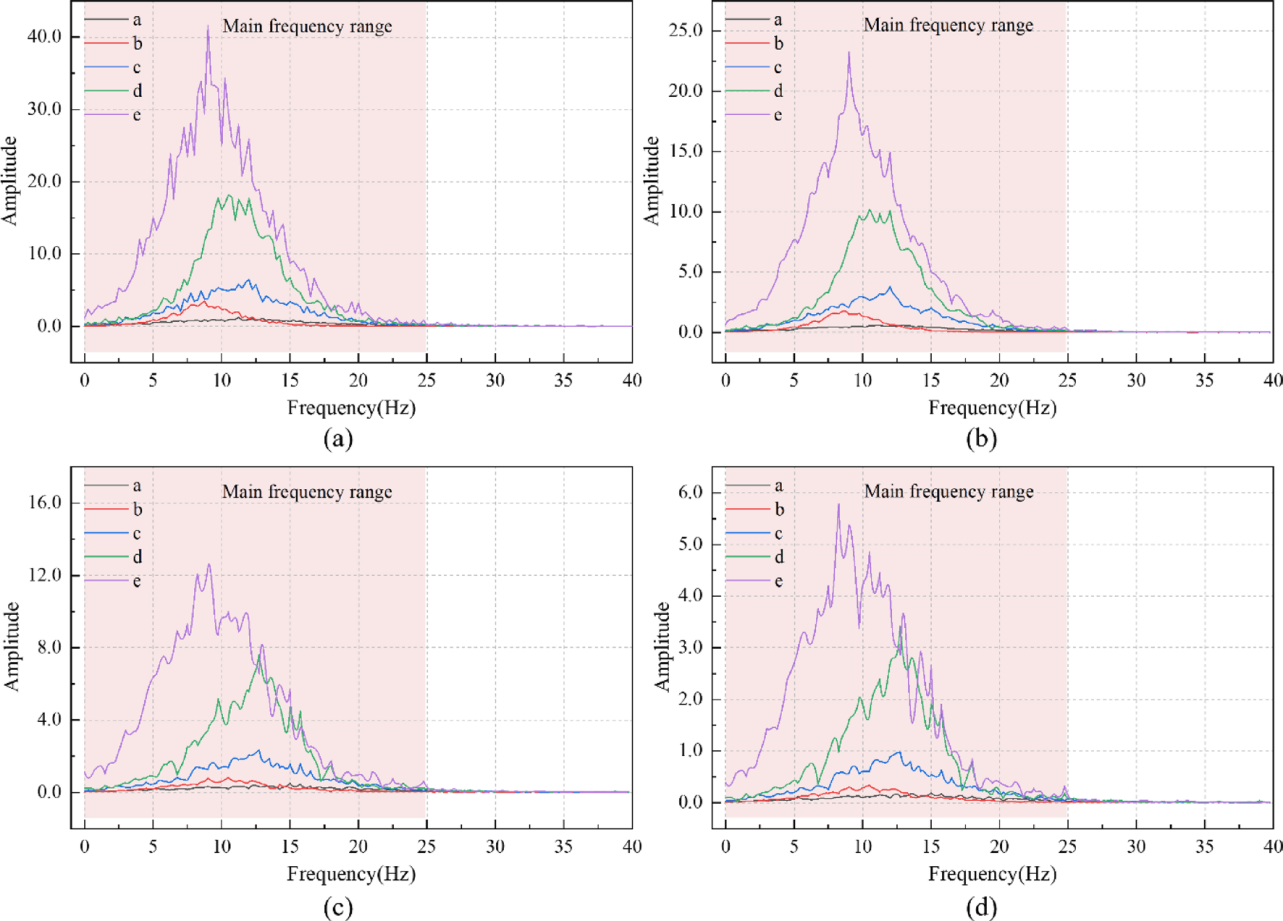
The marginal spectrum of each monitoring point under different working conditions: (**a**) 71 km/h Freight train; (**b**) 74 km/h Passenger train; (**c**) 101 km/h Freight train; (**d**) 104 km/h Passenger train.

To further investigate the energy transfer characteristics of the vibration waves generated by the train loading of the existing line in the structure of the rotating bridge, the marginal spectra of each measurement point of the rotating bridge are analysed as shown in Fig. 17. The frequency of the Fourier transformed time spectra in Figs. 8 and 10 are distributed from 0–40 Hz and show a non-stationary pattern with complex frequency components. In Fig. 17, the main frequencies of the marginal spectra are concentrated in the range of 0–25 Hz and are more regular compared to the former, which more accurately reflect the actual frequency components of the nonsmoothed signal. Under the low-speed operation condition, the train is realised as a single peak at the girder end of the transition bridge (e measurement point); under the high-speed operation condition, the train is realised as a multi-peak at the girder end of the transition bridge (e measurement point). The speed of the existing train will have a significant effect on the energy of the vibration wave, and the lower speed will cause the energy to be concentrated in the vicinity of the higher frequency.

**Figure 18:**
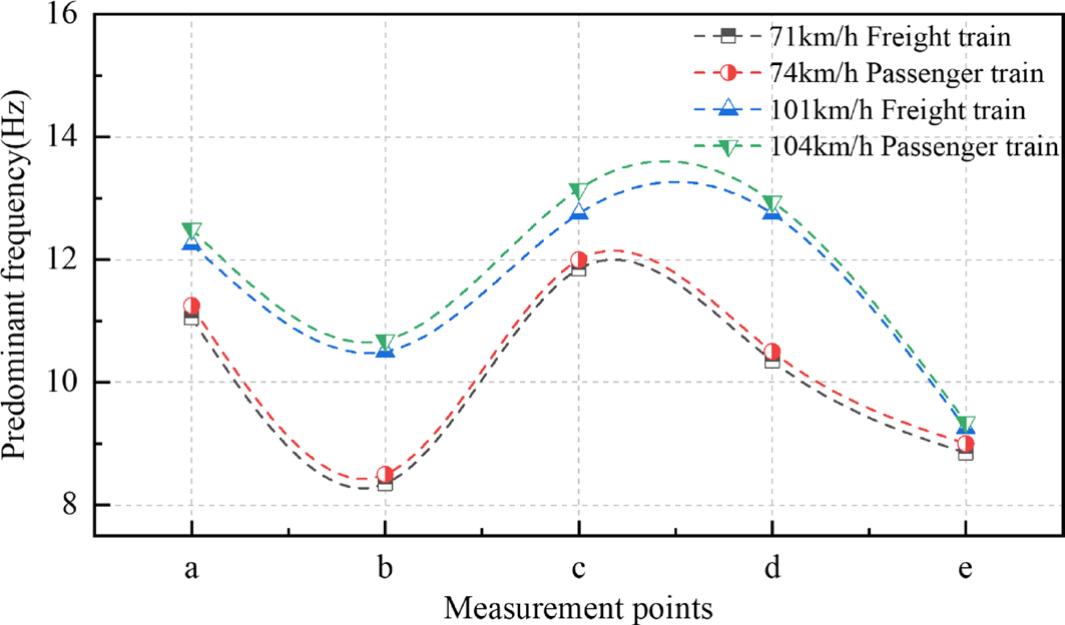
Marginal spectrum predominant frequency variation curve of each measuring point under different working conditions.

By extracting the predominant frequency of different measuring points under different working conditions, the marginal spectrum superior frequency change curve was drawn, as shown in Fig. 18. As can be seen from Fig. 18, the predominant frequency of vibration waves at each measuring point along the transmission path shows a trend of decreasing → increasing → decreasing. The energy of the vibration wave on the beam body of rotating bridge is transferred from high-frequency component to low-frequency component. The predominant frequency of each measuring point shows that: under the same type of train, the low-speed train is smaller; At the same speed, freight trains are lower than passenger trains.

**Figure 19:**
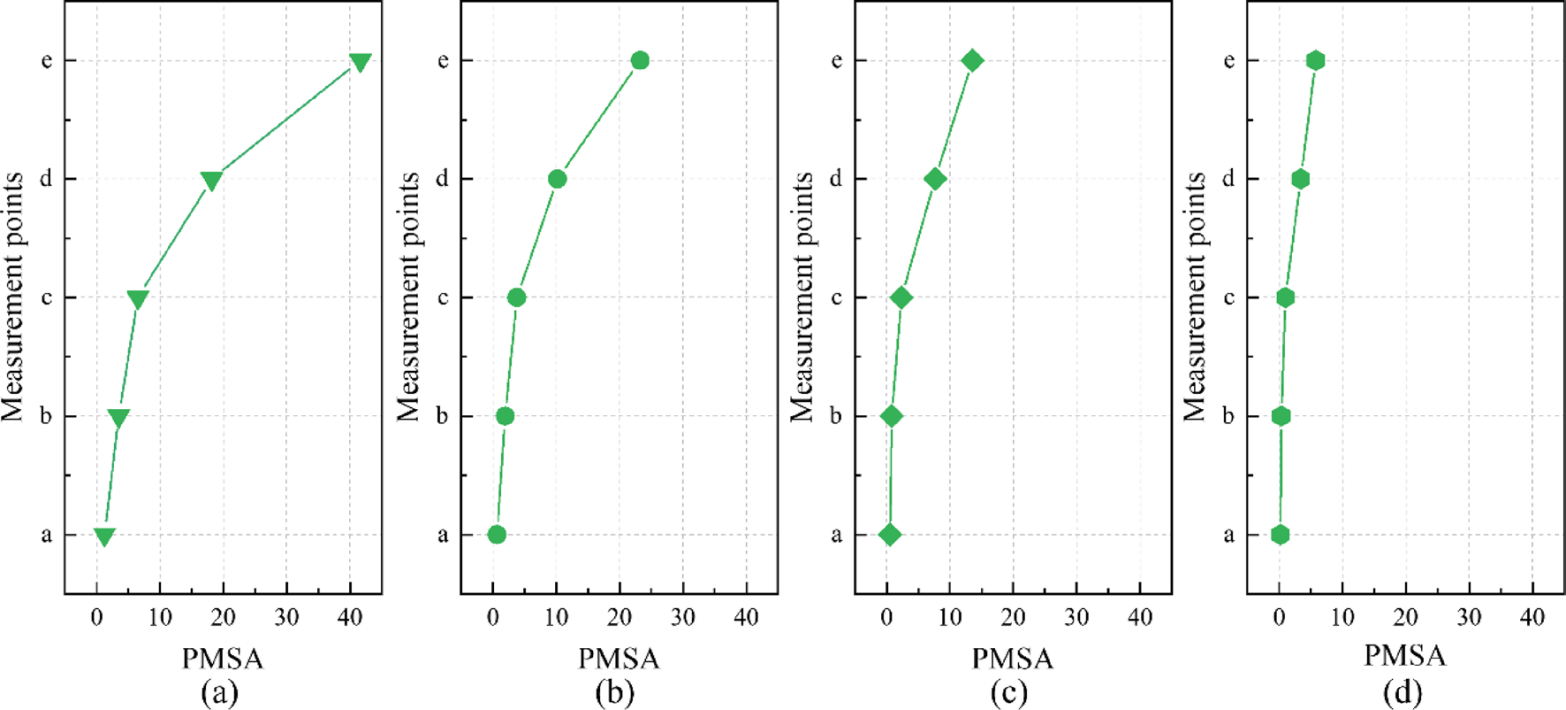
Variation of peak marginal spectrum amplitude (PMSA) under different working conditions: (**a**) 71 km/h Freight train; (**b**) 74 km/h Passenger train; (**c**) 101 km/h Freight train; (**d**) 104 km/h Passenger train.

The Peak Marginal Spectrum Amplitudes (PMSA) at different measurement points under different operating conditions were extracted and the PMSA variation chart was plotted, as shown in Fig. 19. From Fig. 19, the PMSA consistently shows an increasing trend along the transmission of the vibration waves through the rotating bridge structure (a → e). In particular, during the transmission phase between the piers of the rotating bridge (a → c), the PMSA remains relatively stable. However, during the transmission phase between the girders of the rotating bridge (c → e), it intensifies, reaching a maximum growth of 6.407 times. At the same speed, the vibration waves generated by freight trains show a more pronounced impact along the transmission path. The influence of existing trains on the rotating bridge structure can be classified as follows 71 km/h freight train > 74 km/h passenger train > 101 km/h freight train > 104 km/h passenger train. In general, vibration waves generated by low-speed freight trains on existing railways have a greater impact on the rotating bridge structure.

## Conclusion

In order to study the spatial response law and vibration wave transmission mechanism of the rotating bridge structure under the action of the train load of the existing line. This paper conducts field experiments and numerical modal analysis. Signal processing and vibration analysis are performed using time–frequency spectra and the Hilbert–Huang Transform (HHT) energy spectra. The main conclusions are as follows:

- According to the modal analysis and field experiment data, *f*_1_ and *f*_3_ are the natural frequencies (vertical frequencies of the bridge) of the rotating bridge structure. Among them, the *f*_1_ (2–5 Hz) frequency band is the main factor influencing the amplification of the acceleration response of the rotating bridge, while the *f*_3_ (10–13 Hz) frequency band plays a secondary role. Furthermore, the frequency band *f*_2_ (7–9 Hz) corresponds to the frequency components induced by the loading of existing railway trains.
- The frequency distribution characteristics of the vibration waves induced by the train loads on the existing line have an important influence on the acceleration response of the main girder of the rotating bridge. Compared to the train speed, the train type has less influence on the frequency distribution characteristics of the vibration wave, which mainly affects the overall amplitude response of the vibration wave.
- The predominant frequency of vibration waves at each measuring point along the transmission path shows a trend of decreasing → increasing → decreasing. The PMSA consistently shows an increasing trend along the transmission of the vibration waves through the rotating bridge structure (a → e).
- The influence of existing trains on the rotating bridge structure can be classified as follows 71 km/h freight train > 74 km/h passenger train > 101 km/h freight train > 104 km/h passenger train. In general, the impact on the rotating bridge structure of vibration waves generated by low-speed freight trains on existing railways is greater.
- This study demonstrates that the method based on time history spectrum and HHT energy spectrum signal processing and vibration analysis has good applicability in the study of the response mechanism of rotating bridge structure. The method enhances the understanding of the dynamic response characteristics of rotating bridges for engineers and researchers, while also serving as an important basis for their design, evaluation and optimization.

## Data Availability

The datasets used and/or analysed during the current study available from the corresponding author on reasonable request.
